# Health professionals’ perceptions of patient safety competencies: psychometric properties of the French version of the H-PEPSS in France and Switzerland

**DOI:** 10.1186/s12909-023-04893-y

**Published:** 2023-11-29

**Authors:** Sylvain Boloré, Laurent Sovet, Nicolas Guirimand

**Affiliations:** 1 School of Health Sciences, Geneva, HES-SO University of Applied Sciences and Arts Western Switzerland, Sylvain Boloré, 47, Avenue de Champel, 1206 Geneva, Switzerland; 2 Interdisciplinary Education and Training Research Centre (CIRNEF), University of Rouen Normandy, Rouen, France; 3 Université Paris Cité and Univ Gustave Eiffel, LaPEA, 92100 Boulogne-Billancourt, France

**Keywords:** Patient Safety, Competencies, Multiprofessional, Curriculum, Evaluation, Psychometrics

## Abstract

**Background:**

Several initiatives have been implemented to develop, manage, and assess patient safety (PS) competencies, which are considered as a serious public health issue across the world. The Health Professional Education in Patient Safety Survey (H-PEPSS) is widely used as a psychometric scale for evaluating perceived PS competencies but has not been validated in French. The purpose of the study was to investigate the main psychometric properties of the French version of the H-PEPSS.

**Methods:**

A total of 449 students enrolled in nursing and physiotherapy schools in France and French-speaking Switzerland completed a self-administered questionnaire. The 38 items of the H-PEPSS were translated into French following a committee approach. The scale’s construct validity was assessed using confirmatory factor analysis. Reliability of the six factors of the H-PEPSS was evaluated using Cronbach α and McDonald’s ω. Measurement invariance across countries and academic majors as well as discriminant validity were also investigated.

**Results:**

After we removed one item, the H-PEPSS 6-factor model demonstrated adequate goodness-of-fit statistics (χ^2^[194] = 316.633, χ^2^/*df* = 1.632, *p* < 0.001, CFI = 0.934, TLI = 0.922, RMSEA = 0.041 [0.033, 0.049], SRMR = 0.044). The total score can be also used as an overall measure of PS competence (χ^2^[203] = 342.251, χ^2^/*df* = 1.686, *p* < 0.001, CFI = 0.925, TLI = 0.915, RMSEA = 0.043 [0.035, 0.051], SRMR = 0.047). One item was removed because of its high multicollinearity with other items. The reliability was deemed satisfactory (Cronbach α ≥ 0.60), except for the “Understanding human and environmental factors” subscale. Consistently, this subscale was often reported with the lowest reliability in previous studies. We confirmed scalar invariance between countries and partial scalar invariance between majors (ΔCFI ≤ 0.01). The heterotrait-monotrait ratio of correlations ranged from 0.63 to 0.91. In our results, country, academic year, and academic satisfaction were frequently the main predictors of self-reported PS competencies.

**Conclusion:**

Perceived PS competencies can be assessed and fairly compared across France and Switzerland and across nursing and physiotherapy students. We discuss the relevance of the introduction of the H-PEPSS in the training pathway of health professions degree courses and the fallout in clinical contexts.

## Background

Ensuring patient safety (PS) emerges as a clear-cut mission for health care professionals. Nevertheless, despite an international consensus emphasizing the need to improve training for caregivers in PS and clinical risk management in health care [1–4], 10% of hospitalized patients experience an adverse event, most of which are deemed preventable [5]. PS refers to preventing and reducing “risks, errors and harm that occur to patients during provision of health care” (§1) [6]. To achieve this, “good intentions” are not enough. Instead, it necessitates the cultivation of specific yet shared competencies among all health care practitioners, such as those in the Canadian Safety Competencies Framework (CSCF) [7, 8]: teamwork; communication; safety, risk, and quality improvement; optimize human and system factors; recognize, respond to, and disclose PS incidents; and culture of safety.

For students learning the basics of good practice and for experienced professionals, training programs need to be designed to foster the development of these competencies. According to Wu and Busch [3], health professions focus mainly on students acquiring techniques and knowledge but attach too little importance to attitudes and skills necessary to practice safely and spur improvements in care. Moreover, many studies have found deficits in several aspects of PS education, notably communication and teamwork, managing safety risks, and recognizing and responding to adverse events [9–14]. Furthermore, integrating PS content into programs requires a multilevel learning process [15] and is made difficult by the lack of available time in curricula [16], the lack of educators trained in PS [16, 17], and the need to mobilize PS content in practical situations, such as simulation [13, 18, 19]. Certainly, general PS guidelines and handbooks have been published and updated over the past decade [20], yet printed educational materials only slightly help improve health care professionals’ practices [21]. These educational tools are insufficient to address a prevalent and preventable phenomenon. Therefore, there arises a need to create and develop tools tailored for health professionals training, considering the cultural aspects of the institutions they are educated in and the diversity of their student profiles [2, 22].

In France and Switzerland, nursing and physiotherapy education programs are based on a competency-based approach. Individual higher education institutions determine how PS should be taught based on national and international standards [14]. In bachelor’s programs, this topic is not specifically taught but is integrated into other courses, a practice commonly observed across various European countries [14]. For example, in France, it is part of courses related to clinical risk management [23, 24] whereas in Switzerland, it is part of courses focusing on teamwork [25]. However, dedicated courses can be offered in postgraduate training programs [26].

Identifying this type of program’s strengths and weaknesses is possible using a measure of self-efficacy among a group of students [27]. Commonly used in Anglo-Saxon countries, this method is seldom applied in French-speaking Europe, where studies on this topic have concerned the culture of PS and involved only medical students [28, 29]. In view of PS education’s importance in the training of health care professionals, it is particularly challenging to assess how effective such programs may be in stimulating the development of PS competencies [30]. As Okuyama et al. [31] noted, “the assessment of safety competencies is a new field of education, and it is clearly difficult to develop reliable and valid assessment tools” (p. 998).

To address this issue, the Health Professional Education in Patient Safety Survey (H-PEPSS) was developed to measure specifically the self-reported PS competencies among a wide range of health professional groups, including students [32]. The full questionnaire comprises 38 items divided into 4 sections. The first section includes 4 items related to clinical safety. The second section is the central part of the H-PEPSS and consists of 23 items used to measure the 6 domains based on the CSCF (mentioned above) [7, 8]. The third section includes 7 items related to health professional education PS. The last section consists of 4 items related to the confidence of speaking up about PS. Each item is assessed using a 5-point Likert-type scale ranging from 1 (*Strongly disagree*) to 5 (*Strongly agree*), with a “don’t know” (DK) answer option. Subscale scores are calculated by averaging the results for each subscale item. The total score is obtained by adding up the subscale scores.

The psychometric properties of the H-PEPSS are well documented. Confirmatory factor analysis (CFA) revealed a well-fitting 6-factor model for the data. In the original version [32], the Comparative Fit Index (CFI) was 0.948, and the Root Mean Square Error of Approximation (RMSEA) stood at 0.055. In the Korean version [33], these values improved to 0.959 and 0.058 respectively; similarly, the Chinese version [34] demonstrated a CFI of 0.98 and an RMSEA of 0.055. The internal consistency measured with Cronbach α was greater than 0.80 for each subscale in the original version [32] and ranged from 0.70 to 0.81 in the Korean version [33]. For the total score, Cronbach α was 0.91 in the Korean version [33], 0.94 in the Italian version [35], and 0.95 in the Chinese version [34].

The H-PEPSS has been used in 21 countries with over 15,000 subjects [36], mainly with nursing students but never with physiotherapy students. With the availability of a French version of this tool, evaluating the impact of current curriculum implementation and educational interventions, regardless of whether they are interprofessional or not, can be accomplished in French-speaking countries.

### Purpose of the study

The purpose of the study was to investigate the main psychometric properties of the French version of the H-PEPSS, including factor validity, reliability, measurement invariance across countries and academic majors, and discriminant validity.

## Methods

### Study design

A validation study was undertaken, divided into two phases: 1) a linguistic translational and cultural validation of the tool; 2) a cross sectional study design for the evaluation of the psychometric properties of the French version of the H-PEPPS.

### Translation and cultural adaptation

In September 2019, the original author of H-PEPSS granted written authorization for the translation and use of the questionnaire. The French version of the H-PEPSS was prepared following a committee approach [37]. Like the original instrument, the French version also includes the full set of 38 questions, divided into 4 sections. The first stage consisted of 2 independent forward translations of the items from the original language to the French language, performed by the first and last authors of the present study. In the second stage, they met to review and refine the initial translated items. A discussion with the author of the original instrument clarified the meaning of item 18 (i.e., “Safe application of health technology”). The third stage involved a bilingual expert committee, which discussed the semantic, idiomatic, experiential, and conceptual equivalences of the original instrument and the translated instrument. The bilingual expert committee comprised 6 members with different backgrounds, educational levels, and positions in health care education. The fourth stage involved 2 certified translators who independently performed forward translations from French to English. Subsequently, the back-translated items were compared with the original items. The final French version of the H-PEPSS was prepared by discussing and integrating comments and suggestions from the previous stage.

### Participants, setting, and data collection

The target population included nursing and physiotherapy students from 4 faculties of health sciences in Northwest France and in the French-speaking part of western Switzerland (i.e., 2 faculties for each country). The deans of each faculty approved the study. The Regional Research Ethics Committee granted ethics approval. All the participants were recruited between December 2019 and April 2020. A convenience sampling strategy was used. Students with similar majors and academic years were targeted across France and Switzerland to increase the comparability of the subsamples. The rule of 10 individuals for 1 item was used to determine the minimum sample size [38]. A paper-and-pencil form developed using evasys (evasys GmbH, Lüneburg, Germany) was offered to students at 3 faculties. These students were invited to participate with a verbal invitation at the end of a course. In the case of the fourth faculty, an online version developed using LimeSurvey (LimeSurvey GmbH, Hamburg, Germany) was chosen because certain students were engaged in clinical training during the survey period. These students were invited to participate via email, and a reminder was sent 1 week after the initial invitation. Prior to completing the survey, each participant received legal information and provided informed consent. There was no time limit for compiling the survey.

In each faculty, a corresponding teacher facilitated the survey by presenting the process, informing the participants, and conducting the survey. To avoid risk of bias, researchers provided training to teachers from the physiotherapy and nursing schools on the data collection protocol and addressed any questions they had. Given the literature on the psychometric validation of the H-PEPSS in various languages and the rigorous translation process undertaken, conducting a pilot study did not seem imperative.

### Data analysis

Data were analyzed using Excel ver. 16.61. and R Statistical Software (v4.3.1; R Core Team 2023). The naniar package (v1.0.0) was employed for conducting Little’s MCAR test using the mcar_test() function. The factor structure was examined using CFAs. Means (M), standard deviations (SD), skewness, and kurtosis of each item were examined. The Lilliefors-corrected Kolmogorov–Smirnov test was also used to assess the normal distribution of the data. Because of the nonnormality item distribution, structural equation modeling (SEM) was performed using full-information maximum likelihood estimation with robust standard errors and a Satorra-Bentler scaled test statistic with Lavaan [39]. As recommended, χ^2^, the χ^2^/*df* ratio, the Tucker–Lewis Index (TLI), the Comparative Fit Index (CFI), the Root Mean Square Error of Approximation (RMSEA), and the Standardized Root Mean Square Residual (SRMR) were reported as goodness-of-fit indices to determine the fit of each model [40–42]. An χ^2^/df ratio less than 3 is a reliable indicator of a good model fit. TLI and CFI are measures of incremental fit indices based on a comparison between a specified model and a restricted baseline model. TLI and CFI values higher than 0.95 indicate good model fit, but values between 0.90 and 0.95 are also acceptable. RMSEA is an absolute fit index which assesses how well the model reproduces the population covariance matrix. RSMEA values below 0.08 indicate adequate model fit, but values below 0.06 suggest a good model fit. SRMR is also a measure of absolute fit that assesses the standardized difference between the observed and predicted covariance matrices. SRMR values below 0.08 indicate a good fit. As Revelle and Zinbarg [43] recommended, we chose McDonald’s ω to assess reliability, in addition to Cronbach α.

Consistent with the previous literature [44], we treated DK responses as missing data. Missing values are often classified into three distinct categories: missing completely at random (MCAR), missing at random (MAR), and missing not at random (MNAR). Lee et al. [45] defined the 3 categories as follows: “MCAR as the assumption that missingness does not depend on observed or missing data; MAR (but not MCAR) as the assumption that missing data are unrelated to unobserved values given the observed data; and MNAR as the negation of MAR, arising if missingness is related to unobserved values given the observed data” (p. 2). The term MAR may seem counterintuitive, considering that the missingness relies on the observed data. Little’s MCAR test is usually used to determine whether data is MCAR [46].

Measurement invariance was examined using multigroup confirmatory factor analysis (MGCFA) to assess to what extent the adjusted model of the H-PEPSS was similar across countries and academic majors. Configural invariance is the most basic level of measurement invariance. It implies that the underlying factor structure is the same across different groups. Metric invariance is a higher level of invariance than configural invariance. It means that not only is the structure the same across groups, but the factor loadings between the items and the underlying structure are also the same across groups. Scalar invariance is the highest level of measurement invariance. In addition to having the same factor structure and equivalent factor loadings, scalar invariance also requires that the intercepts of the items are the same across groups. Nested model comparisons used for testing metric invariance and scalar invariances were based on the cutoff value of ΔCFI ≤ 0.01 [47].

As Henseler et al. [48] recommended, the discriminant validity was examined using the heterotrait-monotrait ratio of correlations (HTMT), which is used to determine how the factors are related to each other. Values lower than 0.85 suggest good discriminant validity.

In addition, hierarchical multiple regression analyses were conducted for each H-PEPSS subscale and the total scale. Sociodemographic (i.e., gender, age, native, country) and academic variables (i.e., academic year, major) were simultaneously included as controlled variables. In the second step, academic satisfaction was entered.

## Results

### Participants’ characteristics

The initial pool of participants included 478 students, of which 88.0% completed the paper-and-pencil form. We excluded 29 profiles because of the large number of missing values (i.e., at least 12 consecutive missing values). The final sample comprised 449 participants (24.7% men and 72.8% women; 2.5% did not specify their gender) aged 19 to 47 (*M* = 23.94, *SD* = 4.23). Of the participants, 80.4% were natives of the country where we originally collected the data. Respectively, 74.4% and 25.6% of the students attended the nursing and physiotherapy degree program. The respondents comprised 120 sophomores (26.7%), 245 juniors (54.6%), and 84 seniors (18.9%). Table 1 provides detailed sociodemographic characteristics across majors and countries.

**Table 1:** Participants’ Sociodemographic Characteristics (*N* = 449)

	France		Switzerland	
	Nursing (*n* = 65) n (%)	Physiotherapy (*n* = 84) n (%)	Nursing (*n* = 269) n (%)	Physiotherapy (*n* = 31) n (%)
*Gender*				
Female	59 (90.8)	41 (48.8)	209 (77.7)	18 (58.1)
Male	6 (9.2)	41 (48.8)	52 (19.3)	12 (38.7)
No answer	0 (0.0)	2 (2.4)	8 (3.0)	1 (3.2)
*Age*				
19–24 years old	51 (78.5)	68 (81.0)	208 (77.3)	19 (61.3)
25–29 years old	7 (10.8)	8 (9.5)	35 (13.0)	9 (29.0)
Above 29 years old	7 (10.8)	8 (9.5)	18 (6.7)	3 (9.7)
No answer	0 (0.0)	0 (0.0)	8 (3.0)	0 (0.0)
*Native of the country*				
Yes	60 (92.3)	81 (96.4)	201 (74.7)	19 (61.3)
No	3 (4.6)	0 (0.0)	54 (20.1)	12 (38.7)
No answer	2 (3.1)	3 (3.6)	14 (5.2)	0 (0.0)
*Academic year*				
Second year	0 (0.0)	0 (0.0)	120 (44.6)	0 (0.0)
Third year	65 (100.0)	0 (0.0)	149 (55.4)	31 (100.0)
Fourth year	0 (0.0)	84 (100.0)	0 (0.0)	0 (0.0)

### Data screening and preliminary analysis

Twenty profiles (4.5%) contained between 1 and 3 missing values (*M* = 0.05, *SD* = 0.26), and 252 profiles (56.1%) contained at least one instance of the DK response (*M* = 1.61, *SD* = 2.27). Item 27 (i.e., “The nature of systems [e.g., aspects of the organization, management or the work environment including policies, resources, communication and other processes] and system failures and their role in adverse events”] and item 38 (i.e., “In clinical settings, discussion around adverse events focuses mainly on system-related issues, rather than focusing on the individual(s) most responsible for the event”) presented a critical proportion of participants (20.3% and 24.3%, respectively) who chose the DK answer option. Overall, the initial dataset contained 747 missing values (less than 5%), with 188 profiles (41.9%) displaying at least one missing value (*M* = 1.66, *SD* = 2.32).

Little’s MCAR test [46] revealed that the data were not MCAR (χ^2^[5891] = 6264.04, *p* < 0.001). A hierarchical multiple regression showed that sociodemographic characteristics accounted for 3% of the variation of number of missing values (*F*[8, 432] = 2.54, *p* = 0.01). More specifically, the regression coefficients were significant across gender, administration mode, and academic year. Consistently, it was plausible to consider that missing values were MAR [49]. We used multiple imputation to handle the missing data [50].

Using the Lilliefors-corrected Kolmogorov–Smirnov test for testing the normal distribution of the data, we found significant values at *p* < 0.001 for the 38 items. All the results suggested nonnormality of the data [51].

### Factor structure and reliability

Except for TLI, we tested the initial theoretical model of the H-PEPSS and found adequate goodness-of-fit indices: χ^2^(215) = 406.917, χ^2^/*df* = 1.893, *p* < 0.001, CFI = 0.908, TLI = 0.891, RMSEA = 0.049 [0.041, 0.056], SRMR = 0.047. Inspection of modification indices led us to remove item 6 (i.e., “Managing inter-professional conflict”) because of its high multicollinearity with other items. The adjusted model reached acceptable thresholds across all the goodness-of-fit indices: χ^2^(194) = 316.633, χ^2^/*df* = 1.632, *p* < 0.001, CFI = 0.934, TLI = 0.922, RMSEA = 0.041 [0.033, 0.049], SRMR = 0.044. The comparison between the initial and the adjusted models revealed significant improvement, with Δχ^2^(21) = 93.626, *p* < 0.001. These results confirmed that the 6-factor model of the H-PEPSS was relevant for our sample. Standardized factor loadings ranged from 0.399 to 0.721 (*Median value* = 0.589) (Table 2). We also examined a second-order model and found satisfactory goodness-of-fit statistics: χ^2^(203) = 342.251, χ^2^/*df* = 1.686, *p* < 0.001, CFI = 0.925, TLI = 0.915, RMSEA = 0.043 [0.035, 0.051], SRMR = 0.047. This suggests that the total score can be used as an overall measure of PS competence.

**Table 2:** Standardized Factor Loadings and Intercepts of the 6 Patient Safety Competencies of the H-PEPSS based on the CSCF (*N* = 449)

	Loadings [95% CI]	Intercepts
*C1 Working in teams with other health professionals*		
5. Team dynamics and authority/power differences	0.500 [0.405, 0.596]	3.962
7. Debriefing and supporting team members after an adverse event or close call	0.468 [0.375, 0.561]	4.165
8. Engaging patients as a central participant in the health care team	0.399 [0.296, 0.503]	4.695
9. Sharing authority, leadership, and decision making	0.587 [0.492, 0.682]	3.955
10. Encouraging team members to speak up, question, challenge, advocate, and be accountable as appropriate to address safety issues	0.634 [0.550, 0.719]	4.009
*C2 Communicating effectively*		
11. Enhancing patient safety through clear and consistent communication with patients	0.667 [0.597, 0.737]	4.543
12. Enhancing patient safety through effective communication with other health care providers	0.705 [0.630, 0.781]	4.423
13. Effective verbal and nonverbal communication abilities to prevent adverse events	0.721 [0.644, 0.797]	4.241
*C3 Managing safety risks*		
14. Recognizing routine situations and settings in which safety problems may arise	0.574 [0.478, 0.669]	3.927
15. Identifying and implementing safety solutions	0.651 [0.559, 0.742]	3.998
16. Anticipating and managing high risk situations	0.672 [0.588, 0.756]	3.579
*C4 Understanding human and environmental factors*		
17. The role of human factors such as fatigue, competence that effect patient safety	0.576 [0.485, 0.667]	3.969
18. Safe application of health technology	0.410 [0.315, 0.504]	4.065
19. The role of environmental factors, such as work flow, ergonomics, resources, that effect patient safety	0.627 [0.536, 0.718]	3.964
*C5 Recognizing and responding to reduce harm*		
20. Recognizing an adverse event or close call	0.591 [0.513, 0.669]	4.087
21. Reducing harm by addressing immediate risks for patients and others involved	0.639 [0.563, 0.715]	4.143
22. Disclosing the adverse event to the patient	0.514 [0.426, 0.601]	3.557
23. Participating in timely event analysis, reflective practice, and planning in order to prevent recurrence	0.602 [0.521, 0.682]	4.087
*C6 Culture of safety*		
24. The ways in which health care is complex and has many vulnerabilities (e.g., workplace design, staffing, technology, human limitations)	0.524 [0.439, 0.608]	3.982
25. The importance of having a questioning attitude and speaking up when you see things that may be unsafe	0.429 [0.326, 0.533]	4.096
26. The importance of a supportive environment that encourages patients and providers to speak up when they have safety concerns	0.528 [0.442, 0.613]	4.091
27. The nature of systems (e.g., aspects of the organization, management, or the work environment, including policies, resources, communication, and other processes) and system failures and their role in adverse events	0.611 [0.525, 0.696]	3.470

All the values are significant at *p* < 0.001

Table 3 provides all the descriptive statistics. Cronbach α and McDonald’s ω lead to similar values. More precisely, the former was 0.88 for the 22-item scale, and it ranged from 0.54 for the “Understanding human and environmental factors” subscale to 0.74 for the “Communicating effectively” subscale (*Median value* = 0.65). Accordingly, only the “Understanding human and environmental factors” subscale showed inadequate reliability.

**Table 3:** Descriptive Statistics of the 6 Patient Safety Competencies of the H-PEPSS (*N* = 449)

	k	M	SD	Min	Max	Skewness	Kurtosis	α	ω
C1	5	4.16	0.54	2.20	5.00	-0.66	0.17	0.64	0.69
C2	3	4.40	0.53	2.33	5.00	-0.67	0.05	0.74	0.74
C3	3	3.83	0.60	1.67	5.00	-0.50	0.61	0.66	0.67
C4	3	4.00	0.58	2.00	5.00	-0.47	0.37	0.54	0.57
C5	4	3.97	0.58	2.25	5.00	-0.26	-0.24	0.67	0.69
C6	4	3.91	0.59	2.25	5.00	-0.36	-0.17	0.60	0.63
Total	22	4.05	0.42	2.36	5.00	-0.33	0.20	0.88	0.89

*Note*. C1 = Working in teams with other health professionals, C2 = Communicating effectively, C3 = Managing safety risks, C4 = Understanding human and environmental factors, C5 = Recognizing and responding to reduce harm, C6 = Culture of safety. All the descriptive statistics are based on mean item scores. *k* Number of items, *M* Mean, *SD* Standard deviations

The form also included 7 items regarding the respondents’ overall perceptions of the qualities of PS issues as part of the curriculum and clinical practices. As an overall measure of academic satisfaction with PS issues, we examined its unidimensionality. The theoretical model showed an adequate fit to our data: χ^2^(14) = 33.479, χ^2^/*df* = 2.391, *p* < 0.001, CFI = 0.946, TLI = 0.919, RMSEA = 0.060 [0.034, 0.087], SRMR = 0.043. Cronbach α was 0.69 and McDonald’s ω was 0.74 for the current study.

### Measurement invariance across countries and academic majors

As Table 4 shows, the level of invariance across countries reached configural, metric, and scalar invariance. In addition, the level of invariance across academic majors reached configural, metric, and partial scalar invariance. For the latter model, it was necessary to allow intercepts from item 8 (i.e., “Engaging patients as a central participant in the healthcare team”) and item 24 (i.e., “The ways in which healthcare is complex and has many vulnerabilities [e.g., workplace design, staffing, technology, human limitations]”) to be estimated freely in the 2 groups. In general, all the models demonstrated acceptable goodness-of-fit statistics or values very close to the thresholds.

**Table 4:** Measurement Equivalence of the H-PEPSS across Countries, Gender, and Academic Majors (*N* = 449)

	χ^2^	df	χ^2^/df	*p*	CFI	TLI	RMSEA	CI 90	SRMR	ΔCFI
*Measurement across countries*										
Configural invariance	562.805	388	1.450	< 0.001	0.913	0.896	0.048	0.039–0.056	0.054	
Metric invariance	569.826	404	1.410	< 0.001	0.916	0.904	0.046	0.037–0.054	0.058	0.003
Scalar invariance	610.279	420	1.453	< 0.001	0.904	0.894	0.048	0.039–0.056	0.060	0.008
*Measurement across academic majors*										
Configural invariance	535.204	388	1.379	< 0.001	0.928	0.914	0.044	0.034–0.052	0.053	
Metric invariance	565.722	404	1.400	< 0.001	0.920	0.908	0.045	0.036–0.053	0.059	0.008
Scalar invariance	609.284	420	1.451	< 0.001	0.907	0.897	0.048	0.039–0.056	0.061	0.013
Partial scalar invariance	595.964	418	1.428	< 0.001	0.911	0.901	0.046	0.038–0.055	0.063	0.009

*Note*. *CFI* comparative fit index, *TLI* Tucker–Lewis index, *RMSEA* root mean square error of approximation, *SRMR* standardized root mean square residual

### Discriminant validity

Heterotrait-monotrait ratio of correlations (HTMT) ratios ranged from 0.63 to 0.91 (*Median value* = 0.68). Only the HTMT ratio between the “Understanding human and environmental factors” subscale and the “Culture of safety” subscale was above the threshold. Table 5 presents the intercorrelations among all the subscales of the H-PEPSS.

**Table 5:** Intercorrelations among the H-PEPSS Subscales (*N* = 449)

	C1	C2	C3	C4	C5	C5	Total
C1	–						
C2	0.50	–					
C3	0.36	0.46	–				
C4	0.38	0.44	0.42	–			
C5	0.45	0.46	0.49	0.47	–		
C6	0.50	0.45	0.36	0.48	0.52	–	
Total	0.76	0.72	0.65	0.68	0.77	0.77	–

*Note*. C1 = Working in teams with other health professionals, C2 = Communicating effectively, C3 = Managing safety risks, C4 = Understanding human and environmental factors, C5 = Recognizing and responding to reduce harm, C6 = Culture of safety. All the descriptive statistics are based on mean item scores. All the correlations are significant at *p* < 0.001

As Table 6 shows, all the models were significant predictors of PS competencies at *p* < 0.001, which explained between 6% (i.e., “Working in teams with other health professionals”) and 18% (i.e., “Understanding human and environmental factors”) of their respective variances. For the total score, 22% of the variance was explained, *F*(7, 426) = 18.12, *p* < 0.001. Total Scale shows that country, academic year, and academic satisfaction were frequently the main predictors. It is noteworthy that academic satisfaction was the most important predictor, adding an incremental variance ranging from 4 to 14% at the subscale level and 16% for the total score.

**Table 6:** Hierarchical Regression Analyses for H-PEPSS Subscales and Total Scale (*N* = 434)

	C1	C2	C3	C4	C5	C6	Total
*Step 1*							
Gender (1 = Male)	0.05	0.08	0.03	0.03	-0.03	0.04	0.04
Age	-0.04	-0.03	0.06	-0.09*	0.08	-0.01	-0.01
Native (1 = Yes)	-0.11*	-0.05	0.05	-0.05	-0.01	-0.08	-0.06
Country (1 = Switzerland)	0.19**	0.19**	0.11	0.19**	0.28***	0.30***	0.29***
Academic Year	0.23**	0.32***	0.26**	0.26***	0.14	0.17*	0.30***
Major (1 = Nursing)	0.01	0.05	-0.07	0.05	-0.10	0.00	-0.02
*Step 2*							
Academic Satisfaction	0.21***	0.35***	0.36***	0.40***	0.31***	0.33***	0.43***
*Adjusted R*^*2*^	0.06	0.15	0.14	0.18	0.13	0.17	0.22
Δ*R*^*2*^ (Step 2 – Step 1)	0.04	0.10	0.11	0.14	0.08	0.09	0.16

*Note*. Standardized regression coefficients (β) are reported. C1 = Working in teams with other health professionals, C2 = Communicating effectively, C3 = Managing safety risks, C4 = Understanding human and environmental factors, C5 = Recognizing and responding to reduce harm, C6 = Culture of safety. For the ordinal variable, the value “1” represents the presence of the specified category

^*^p < 0.05, ** *p* < 0.01, *** *p* < 0.001

## Discussion

The aim of this study was to develop a French version of the H-PEPSS and to evaluate its psychometric properties in a sample of nursing and physiotherapy students from France and Switzerland. Our results indicate that the French version of the H-PEPSS in this sample demonstrates good psychometric properties, including good construct validity, internal consistency, and measurement invariance across countries and academic majors. Additionally, the discriminant validity of the French H-PEPSS was also supported, and sociodemographic and academic variables predicted PS competences.

Consistent with previous adaptation studies [34, 35, 52, 53], our results revealed we can make a good tool that fully replicated the original factor structure. An adjusted model featuring 22 items, achieved by omitting the “Managing inter-professional conflict” item present in the original version, met acceptable thresholds for all goodness-of-fit indices. In addition, a second-order model displayed satisfactory goodness-of-fit indices, suggesting that the total score can also be used as an overall measure of PS competence. The internal consistency of the French H-PEPSS, in terms of Cronbach α, was 0.88 for the total score and ranged from 0.54 to 0.74 for the 6 domains. Only the “Understanding human and environmental factors” subscale showed inadequate reliability. In contrast with the original study [32], this subscale was often reported as having the lowest reliability in previous studies [33–35, 52]. In addition, it demonstrated scalar invariance across France and Switzerland and partial scalar invariance across majors (ΔCFI ≤ 0.01). This observation suggests that students majoring in nursing and physiotherapy might attribute varied interpretations to the concept of “health care.” In general, these results suggest that perceived PS competencies can be assessed and fairly compared across France and Switzerland and across nursing and physiotherapy students. In terms of discriminant validity, HTMT ranged from 0.63 to 0.91. Only the HTMT ratio between the “Understanding human and environmental factors” subscale and the “Culture of safety” subscale exceeded the threshold, possibly because both subscales refer to organizational aspects.

Additionally, our hierarchical regression analyses for H-PEPSS subscales and Total Scale shows country, academic year, and academic satisfaction were frequently the main predictors of PS competencies. In other terms, these variables were more likely to significantly predict the level of perception in each competency as well as the total score. These results provide new insights since no previous study examined the role of these variables among nursing and physiotherapy students in France and Switzerland. In a previous study conducted in China [54], the authors found that 15% of the total variance in PS competencies was explained by PS learning styles (self-study and classroom study), by different self-assessed PS competence levels, and by experiences of adverse events (*p* < 0.05).

The French H-PEPSS’ version is currently the only one available in French to measure the perceptions of health care professionals’ PS knowledge and competence. We consider that this scale is an interesting tool to reinforce students’ critical thinking about their perception of their practice through a formative evaluation that is necessary for all learning [55]. Its use could be associated with a summative assessment aimed at evaluating students’ performance in terms of safety of care during their practice or interprofessional education course [33].

The H-PEPSS has been adapted in various languages and countries. The comparison of the psychometric properties of this scale translated in each of these countries is made difficult for several reasons. Firstly, there is a variability of the number of items retained by each country. Whereas researchers in some countries retain all items from the original Canadian version (e.g., Chinese or French), others retain, for example, only 23 [35] or 15 items in a short version [33], possibly affecting measures of composite reliability. Secondly, the status (i.e., physicians, surgeons) and number of subjects who completed the questionnaires also varied greatly from one study to another. In some studies, the questionnaire was validated exclusively with student nurses [30, 34, 35, 52], whereas in the original version, it included nurses, physicians, and pharmacists [9]. Our study included student nurses and physiotherapists.

Because Cronbach α can underestimate or overestimate reliability [56, 57], to measure the French scale’s composite reliability, we also used McDonald’s ω, whose validity does not require the items to be true-score equivalent but needs the items to be homogeneous [58, 59]. Both lead to similar values. Last, it is interesting to note that in Ginsburg’s [32] factor analysis, 7 items were removed from the 23 questions of the 6 competency domains (i.e., items 5 to 27).

The H-PEPSS also differs from the Latino Student Patient Safety Questionnaire [60] and from the scale by Flin et al. [61] on medical undergraduates’ knowledge of and attitudes toward medical error. Whereas the H-PEPSS was used to measure health professionals’ perceptions of their PS competence, these other two instruments were designed to measure educational needs for future physicians and nurses and their attitudes. The comparison of these various types of assessment should enlighten students on the performance of their care practice, particularly in terms of avoidable risks, by leading them to reflect on the value of communication between healthcare professionals, whatever the hierarchical distance that separates them.

### Limitations

The limitations of the self-assessment of competence tools derive from the tool’s purpose: it only measures the student’s perception, close to what Bandura [62] calls *self-efficacy*. Although students’ perception of their competence influences their behavior in general [63] and in particular in care situations in various ways, the evaluation of a negative perception would enable trainers to identify the students most prone to difficulties in practical situations. Wood and Bandura [64] found that the perception of one’s competence influences the intensity of the effort made as well as that effort’s quality and effectiveness. Individuals who perceive themselves as competent are more effective in problem analysis than those who doubt their abilities. This problem analysis skill (i.e., reflexive attitude) has become a central element in the diagnosis of healthcare professionals (e.g., nurses, physiotherapists).

It does not by itself prevent possible biases, such as the Dunning-Kruger effect [65], and it does not make it possible to assess precisely whether the student’s perception is close to his or her real professional competence in a work situation or, on the contrary, to underestimate his or her incompetence.

Furthermore, despite the authors’ efforts to produce a faithful translation of the initial tool by adapting it to the usual vocabulary used in training courses for care professionals, potential nuances or cultural subtleties embedded in the source language may not have been fully captured. This could potentially lead to minor variations in the interpretation and response patterns among participants using the translated tool.

## Conclusions

The study examined the psychometric properties of the French version of the H-PEPSS among nursing and physiotherapy students from France and Switzerland. Results showed that the French H-PEPSS had good construct validity, internal consistency, and measurement invariance across countries and academic majors. The discriminant validity of the French H-PEPSS was also supported, and sociodemographic and academic variables predicted PS competences. This tool provides health care educators with guidance for adapting educational interventions, whether interprofessional or not, and can be used to promote a reflective attitude among students.

Healthcare Professionals are confronted with complex clinical situations and problems. This tool would allow them to monitor their progress in terms of clinical reasoning, to limit confused analyses of situations that put patients at risk, through training in problem solving and self-efficacy. This tool offers health care educators avenues of intervention which can promote a better perception of the skills to be acquired and used in acquiring and using theoretical knowledge (declarative knowledge) and practical knowledge (procedural and conditional knowledge). The use of this scale can be seen as an opportunity for students to make sense of their practice and to adopt a reflective attitude. This supports structuring in learning, especially in a context of health crisis where there is an intense feeling among students of being neglected in training. Further studies would be required to generalize the conclusions to other French-speaking countries and healthcare majors.

## Data Availability

All data sets and materials are available from the corresponding author.

## Abbreviations

CFA: Confirmatory factor analysis
CFI: Comparative fit index
DK: Don’t know
H-PEPSS: Health Professional Education in Patient Safety Survey
HTMT: Heterotrait-monotrait ratio of correlations
M: Mean
MAR: Missing at random
MCAR: Missing completely at random
MGCFA: Multigroup confirmatory factor analysis
MNAR: Missing not at random
N: Number of participants
PS: Patient safety
RMSEA: Root mean square error of approximation
SD: Standard deviation
SEM: Structural equation modeling
SRMR: Standardized root mean square residual
TLI: Tucker–Lewis index

## References

[1] World Health Organization. Patient safety curriculum guide: multi-professional edition. Accessed 27 Apr 2023. https://www.who.int/publications/i/item/9789241501958

[2] Aiken LH, Cimiotti JP, Sloane DM, Smith HL, Flynn L, Neff DF (2011). Effects of nurse staffing and nurse education on patient deaths in hospitals with different nurse work environments. Med Care.

[3] Wu AW, Busch IM (2019). Patient safety: a new basic science for professional education. GMS J Med Educ..

[4] Abildgren L, Lebahn-Hadidi M, Mogensen CB (2022). The effectiveness of improving healthcare teams’ human factor skills using simulation-based training: a systematic review. Adv Simul.

[5] Waterson P (2014). Patient safety culture: theory, methods and application.

[6] World Health Organization. Patient safety. Accessed 27 Apr 2023. https://www.who.int/news-room/fact-sheets/detail/patient-safety

[7] Frank JR, Brien S. The safety competencies: enhancing patient safety across the health professions. Canadian Patient Safety Institute; 2008.

[8] Canadian Patient Safety Institute. The safety competencies: enhancing patient safety across the health professions. 2nd ed. Edmonton; 2020. Accessed 17 Aug 2023. https://www.healthcareexcellence.ca/media/115mbc4z/cpsi-safetycompetencies_en_digital-final-ua.pdf

[9] Ginsburg LR, Tregunno D, Norton PG (2013). Self-reported patient safety competence among new graduates in medicine, nursing and pharmacy. BMJ Qual Saf.

[10] Doyle P, VanDenKerkhof EG, Edge DS, Ginsburg L, Goldstein DH (2015). Self-reported patient safety competence among Canadian medical students and postgraduate trainees: a cross-sectional survey. BMJ Qual Saf.

[11] Duhn L, Karp S, Oni O, Edge D, Ginsburg L, VanDenKerkhof E (2012). Perspectives on patient safety among undergraduate nursing students. J Nurs Educ.

[12] Usher K, Woods C, Parmenter G (2017). Self-reported confidence in patient safety knowledge among Australian undergraduate nursing students: a multi-site cross-sectional survey study. Int J Nurs Stud.

[13] Pereira FSH, Garcia DB, Ribeiro ER (2022). Identifying patient safety competences among anesthesiology residents: systematic review. Braz J Anesthesiol.

[14] Kirwan M, Riklikiene O, Gotlib J, Fuster P, Borta M (2019). Regulation and current status of patient safety content in pre-registration nurse education in 27 countries: findings from the rationing - missed nursing care (RANCARE) COST action project. Nurse Educ Pract.

[15] Walton M, Woodward H, Staalduinen S (2010). The WHO patient safety curriculum guide for medical schools. BMJ Qual Saf.

[16] Tregunno D, Ginsburg L, Clarke B, Norton P (2014). Integrating patient safety into health professionals’ curricula: a qualitative study of medical, nursing and pharmacy faculty perspectives. BMJ Qual Saf.

[17] Ladden MD, Bednash G, Stevens DP, Moore GT (2006). Educating interprofessional learners for quality, safety and systems improvement. J Interprof Care.

[18] Cooper S, Cant R, Porter J (2012). Simulation based learning in midwifery education: a systematic review. Women Birth.

[19] Norman J (2012). Systematic review of the literature on simulation in nursing education. J Assoc Black Nurs Facult.

[20] Lachman P, Runnacles J, Jayadev A, Brennan J, Fitzsimons J. Oxford Professional Practice: Handbook of Patient Safety. Oxford University Press; 2022. Accessed 7 Aug 2023. https://global.oup.com/academic/product/oxford-professional-practice-handbook-of-patient-safety-9780192846877?cc=fr&lang=en&

[21] Giguère A, Zomahoun HTV, Carmichael PH (2020). Printed educational materials: effects on professional practice and healthcare outcomes. Cochr Database Syst Rev.

[22] Aiken LH, Clarke SP, Cheung RB, Sloane DM, Silber JH (2003). Educational levels of hospital nurses and surgical patient mortality. JAMA.

[23] Jean S (2019). Introductory remarks on the urgency of physiotherapy. Kinésithérapie, la Revue.

[24] Vignier N. Soins infirmiers et gestion des risques - soins éducatifs et préventifs - Qualité des soins et évaluation des pratiques. Elsevier Health Sciences; 2013. Accessed 16 Aug 2023. https://www.elsevier-masson.fr/soins-infirmiers-et-gestion-des-risques-soins-educatifs-et-preventifs-qualite-des-soins-et-evaluation-des-pratiques-9782294757693.html

[25] Boloré S, Terraneo F (2020). Training in the clinical nursing examination and interprofessionality. Soins cadres.

[26] Schwendimann R, Fierz K, Spichiger E, Marcus B, De Geest S (2019). A master of nursing science curriculum revision for the 21st century – a progress report. BMC Med Educ.

[27] Eva KW, Regehr G (2005). Self-assessment in the health professions: a reformulation and research agenda. Acad Med..

[28] Chaneliere M, Jacquet F, Occelli P, Touzet S, Siranyan V, Colin C (2016). Assessment of patient safety culture: what tools for medical students?. BMC Med Educ.

[29] Larramendy-Magnin S, Anthoine E, L’Heude B, Leclère B, Moret L (2019). Refining the medical student safety attitudes and professionalism survey (MSSAPS): adaptation and assessment of patient safety perception of French medical residents. BMC Med Educ.

[30] Alquwez N, Cruz JP, Alshammari F (2019). A multi-university assessment of patient safety competence during clinical training among baccalaureate nursing students: a cross-sectional study. J Clin Nurs.

[31] Okuyama A, Martowirono K, Bijnen B (2011). Assessing the patient safety competencies of healthcare professionals: a systematic review. BMJ Qual Saf.

[32] Ginsburg L, Castel E, Tregunno D, Norton PG (2012). The H-PEPSS: an instrument to measure health professionals’ perceptions of patient safety competence at entry into practice. BMJ Qual Saf.

[33] Hwang JI, Yoon TY, Jin HJ, Park Y, Park JY, Lee BJ (2016). Patient safety competence for final-year health professional students: perceptions of effectiveness of an interprofessional education course. J Interprof Care.

[34] Chen L, Huang F, Yuan X, Song J, Chen L (2019). An assessment of the reliability and factorial validity of the Chinese version of the Health Professional Education in Patient Safety Survey (H-PEPSS). Front Psychol.

[35] Bressan V, Stevanin S, Bulfone G, Zanini A, Dante A, Palese A (2016). Measuring patient safety knowledge and competences as perceived by nursing students: an Italian validation study. Nurse Educ Pract.

[36] Boloré S. Annexe A - État des lieux de l’usage du questionnaire H-PEPSS. In: From Didactic-Pedagogical Configuration to Organizational Learning Potential: Analysis of an Interprofessional Simulation and Its Impact on Patient Safety Competencies. PhD Thesis. Normandie University; 2022.

[37] Sousa VD, Rojjanasrirat W (2010). Translation, adaptation and validation of instruments or scales for use in cross-cultural health care research: a clear and user-friendly guideline. J Eval Clin Pract.

[38] Kyriazos TA (2018). Applied psychometrics: sample size and sample power considerations in factor analysis (EFA, CFA) and SEM in general. Psychology.

[39] Rosseel Y (2011). lavaan: An R package for structural equation modeling. J Stat Softw..

[40] Hu LT, Bentler PM (1998). Fit indices in covariance structure modeling: sensitivity to underparameterized model misspecification. Psychol Methods.

[41] Kline RB. Principles and practice of structural equation modeling. The Guilford Press; 1998.

[42] Sun J (2005). Assessing goodness of fit in confirmatory factor analysis. Meas Eval Couns Dev.

[43] Revelle W, Zinbarg RE (2009). Coefficients alpha, beta, omega, and the glb: comments on Sijtsma. Psychometrika.

[44] Denman DC, Baldwin AS, Betts AC, McQueen A, Tiro JA (2018). Reducing “I don’t know” responses and missing survey data: implications for measurement. Med Decis Making.

[45] Lee KJ, Carlin JB, Simpson JA, Moreno-Betancur M (2023). Assumptions and analysis planning in studies with missing data in multiple variables: moving beyond the MCAR/MAR/MNAR classification. Int J Epidemiol.

[46] Little RJA (1988). A test of missing completely at random for multivariate data with missing values. J Am Stat Assoc.

[47] Chen FF (2007). Sensitivity of goodness of fit indexes to lack of measurement invariance. Struct Equ Modeling.

[48] Henseler J, Ringle CM, Sarstedt M (2015). A new criterion for assessing discriminant validity in variance-based structural equation modeling. J Acad Mark Sci.

[49] Fox-Wasylyshyn SM, El-Masri MM (2005). Handling missing data in self-report measures. Res Nurs Health.

[50] Donders ART, van der Heijden GJMG, Stijnen T, Moons KGM (2006). Review: a gentle introduction to imputation of missing values. J Clin Epidemiol.

[51] Ghasemi A, Zahediasl S (2012). Normality tests for statistical analysis: a guide for non-statisticians. Int J Endocrinol Metab.

[52] Taskiran G, Eskin Bacaksiz F, Harmanci Seren AK (2020). Psychometric testing of the Turkish version of the Health Professional Education in Patient Safety Survey: H-PEPSSTR. Nurse Educ Pract.

[53] Bergs J, Peeters K, Kortleven I (2021). Translation and validation of the Dutch version of the health professional education in patient safety survey amongst nursing students in Belgium: a psychometric analysis. PLoS ONE.

[54] Huang F, Shen XY, Chen XL, He L, Huang SF, Li JX. Self-reported confidence in patient safety competencies among Chinese nursing students: a multi-site cross-sectional survey. BMC Med Educ. 2020;20(1). 10.1186/s12909-020-1945-8 10.1186/s12909-020-1945-8 PMC6995154 32005224

[55] Black P, Wiliam D (2010). Inside the black box: raising standards through classroom assessment. Phi delta kappan.

[56] Raykov T (1997). Scale reliability, Cronbach’s coefficient alpha, and violations of essential tau-equivalence with fixed congeneric components. Multivariate Behav Res.

[57] Raykov T (2001). Bias of coefficient afor fixed congeneric measures with correlated errors. Appl Psychol Meas.

[58] McDonald RP. Test theory: a unified treatment. Psychology Press; 1999. 10.4324/9781410601087

[59] Raykov T, Shrout PE (2002). Reliability of scales with general structure: point and interval estimation using a structural equation modeling approach. Struct Equ Model.

[60] Mira JJ, Navarro IM, Guilabert M (2015). A Spanish-language patient safety questionnaire to measure medical and nursing students’ attitudes and knowledge. Rev Panam Salud Publica.

[61] Flin R, Patey R, Jackson J, Mearns K, Dissanayaka U (2009). Year 1 medical undergraduates’ knowledge of and attitudes to medical error. Med Educ.

[62] Bandura A (1983). Self-efficacy determinants of anticipated fears and calamities. J Pers Soc Psychol.

[63] Schunk DH (1989). Self-efficacy and achievement behaviors. Educ Psychol Rev.

[64] Wood R, Bandura A (1989). Impact of conceptions of ability on self-regulatory mechanisms and complex decision making. J Pers Soc Psychol.

[65] Kruger J, Dunning D (1999). Unskilled and unaware of it: how difficulties in recognizing one’s own incompetence lead to inflated self-assessments. J Pers Soc Psychol.

